# Identification of key genes for mandibular prognathism in Duolang sheep via genome-wide association analysis

**DOI:** 10.3389/fvets.2025.1719178

**Published:** 2026-01-19

**Authors:** Chao Fang, Ling-Ling Liu, Wu-Jun Liu, Frederic Farnir

**Affiliations:** 1 College of Animal Science, Xinjiang Agricultural University, Urumqi, China; 2 Faculté de Médecine Vétérinaire, Université de Liège Quartier Vallée, Liège, Belgium

**Keywords:** candidate genes, Duolang sheep, enrichment analysis, GWAS, mandibular prognathism

## Abstract

Mandibular prognathism is a craniofacial trait that negatively affects feed intake in sheep and thus influences body weight and production performance. A genome-wide association study (GWAS) was conducted to identify genetic variants associated with mandibular prognathism (MP) in Duolang sheep. Phenotypes were classified into a binary trait: normal and MP. The analysis was based on whole-genome resequencing data from 221 individuals. We identified 48 potentially associated SNPs and 77 candidate genes related to mandibular prognathism. Based on gene functional analysis, we found five genes (PAX7, PLXNA4, CHD2, DZIP1, FBXW7) that may be involved in mandibular prognathism.Notably, seven SNPs on chromosome 4 were located within the PLXNA4 gene, and seven SNPs on chromosome 17 were located upstream of the FBXW7 gene. These findings provide novel insights into the genetic architecture of mandibular prognathism in sheep and offer potential molecular targets for future breeding and selection programs.

## 1 Introduction

Mandibular prognathism (MP), commonly referred to as underbite, is a craniofacial developmental anomaly characterized by anterior displacement of the mandible relative to the cranial base, resulting in malocclusion (1, 2). Clinically, according to Angle’s classification, it is categorized as class III malocclusion, in which the mesiobuccal cusp of the upper first molar occludes distal to the mesiobuccal groove of the lower first molar (3). MP may occur with or without maxillary hypoplasia (4). This disproportionate mandibular overgrowth relative to the craniofacial skeleton, historically termed the “Habsburg jaw,” which accounts for approximately 63–73% of all class III malocclusions, can cause functional impairment and reduced feeding efficiency in both humans and animals (5, 6).

The prevalence of MP shows marked variation across populations and species. In humans, it is most frequent among Asian populations (up to 15%), moderate in African populations (10–16.8%), and relatively rare among Caucasians (1%) (7). Population-level variation suggests a substantial genetic contribution. Environmental influences likely modulate dental/occlusal features rather than basal mandibular length. In livestock, jaw deformities have also been documented: for instance, the incidence of brachygnathia inferior in Brown Swiss cattle ranges from 0.1–5% (8), while an incidence of 29.5% of mandibular prognathism has been reported in Swiss Black Brown Mountain sheep (9). In wild ruminants, the frequency is typically below 1% (10). These differences highlight the complex genetic and environmental contributions underlying MP.

Research on craniofacial malformations in sheep has been sparse and mainly limited to descriptive and morphometric studies. Previous reports have largely focused on mandibular shortening in East Friesian sheep and their crossbreds, where affected individuals displayed distinct brachygnathia inferior phenotypes (10–12). However, no systematic investigations of mandibular prognathism have been conducted in native sheep breeds from China.

The Duolang sheep breed is one such population and generally produces one lamb per ewe. It is mainly distributed in Maigaiti, Bachu, Yuepuhu, and Shache counties of Xinjiang Province, where it has evolved specific adaptations to arid desert conditions (13). Despite its economic importance, genetic studies in Duolang sheep remain limited. In recent years, genome-wide association studies (GWAS) in sheep have predominantly focused on economically important traits, such as growth and body size, reproductive performance, wool and cashmere quality, and health-related resilience. For instance, Li et al. identified candidate genes associated with growth traits in Hu sheep (14), while Liu et al. reported loci related to body-size traits in Tibetan sheep (15). Beyond production-related traits, GWAS has also been extended to longevity and reproductive traits (16). Nevertheless, systematic GWAS addressing craniofacial abnormalities, particularly mandibular prognathism, remain extremely scarce in sheep. Here, we conducted a GWAS comparing normal and MP phenotypes in sheep from Kashi, Xinjiang, to identify candidate genes associated with this trait. Our findings provide new insights into the genetic basis of MP, offering potential targets for future therapeutic interventions and genetic improvement strategies.

## 2 Materials and methods

### 2.1 Sample collection and phenotyping

**Figure 1:**
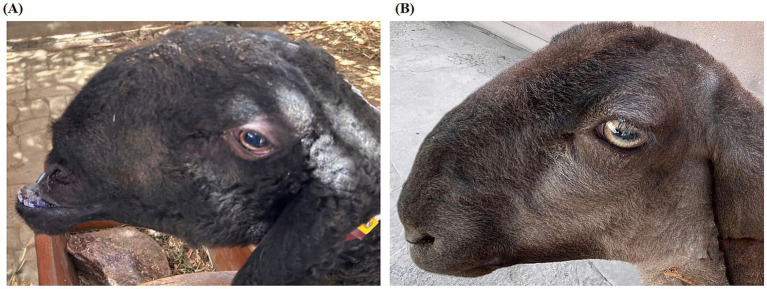
Phenotypic characteristics of Duolang sheep. **(A)** MP phenotype. **(B)** Normal phenotype. Reproduced from (61), licensed under CC-BY-4.0.

A total of 242 blood samples were collected from sheep in Kashi, Xinjiang, China. Each animal was screened for mandibular prognathism (MP, commonly referred to as underbite). The trait was coded as binary (1 = affected; 0 = unaffected). Phenotypic features are shown in Figure 1. MP was assessed in the field using a simple, standardized procedure: one handler restrained the head, while a trained examiner checked the occlusion in natural intercuspation with a neutral head position. A case was recorded when the lower incisors lay anterior to the upper incisors on direct inspection, consistent with the study criteria, while upper and lower incisors were aligned in controls. Husbandry practices were routine and comparable between groups. No targeted nutritional interventions were reported or used. Sampling took place in the morning and was performed by the same team following the standard protocol. Due to oversight by the sampling team, the phenotypes of 21 individuals could not be determined; thus, 221 individuals were included in the GWAS analysis.

### 2.2 DNA extraction and sequencing

Genomic DNA was extracted using a standard phenol-chloroform protocol. DNA quality was evaluated via 1% agarose gel electrophoresis, and concentrations were quantified using a Qubit fluorometer (Invitrogen, Carlsbad, CA, USA). Purified DNA samples were shipped on dry ice to Novogene Bioinformatics Institute (Beijing, China). Following fragmentation, adapter ligation, and PCR amplification, 350-bp libraries were constructed for each individual. Sequencing was performed on an Illumina NovaSeq 6,000 platform (Illumina Inc., San Diego, CA, USA) in 2 × 150-bp paired-end mode. Libraries were sequenced to a target depth of ~10 × per individual.

### 2.3 Read mapping and variant detection

Sequenced reads were processed using Trimmomatic v0.39 (17) with the parameters “LEADING:20 TRAILING:20 SLIDINGWINDOW:4:20 MINLEN:90” to remove low-quality bases. The filtered reads were aligned to the reference genome assembly (ARS-UI_Ramb_v3.0) using the BWA-MEM algorithm v0.7.17-r1188 (18) with default settings. The resulting alignment files were coordinate-sorted by chromosome using Samtools v1.17 (19), duplicates were marked using the MarkDuplicates module in GATK v4.4.0.0 (20), and mate-pair information was synchronized using GATK’s FixMateInformation module.

Both raw and clean sequencing reads were evaluated using Seqkit v2.6.127 (21) and FastQC v0.11.928 to assess base counts and quality metrics. Variant calling was performed for each individual using GATK’s HaplotypeCaller module, followed by merging variant files with the CombineGVCFs module. Joint genotyping was conducted with the GenotypeGVCFs module. Hard filtering criteria were applied using the VariantFiltration module, with thresholds as follows: for SNPs, “QD<3.0 || FS>30.0 || SOR > 4.0 || MQ<30.0 || MQRankSum<-10.0 || QUAL<50.0 || ReadPosRankSum<-5.0,” for InDels, “QD<3.0 || FS>100.0 || ReadPosRankSum <-10.0.” Subsequently, population-level filtering was applied using VCFtools v0.1.17 (22) to ensure data quality for downstream analyses. This step involved the removal of variants with excessive missing data (missing genotype rate > 20%, parameter: --max-missing 0.8) and those with a minor allele frequency (MAF) below 0.03 (−-maf 0.03). This filtering pipeline identified a total of 35,663,272 SNPs and 4,161,247 InDels.

The preliminarily quality-controlled VCF files were converted to binary format using PLINK v1.9 (23). Further quality control for autosomal SNPs was performed with PLINK v1.9. The following criteria were applied: individual call rate ≥ 80%; SNP call rate ≥ 80%; minor allele frequency ≥ 0.03; and Hardy–Weinberg equilibrium *p*-value ≥ 1 × 10^−5^.

### 2.4 PCA analysis

PCA was conducted using PLINK v1.9. To reduce linkage disequilibrium, SNPs were pruned with the command: “--indep-pairwise 50 5 0.2.” Pruned markers were extracted and used to compute the top 20 principal components. The resulting eigenvectors were visualized using R v4.5.0 to assess population structure.

### 2.5 Genome-wide association study (GWAS)

GWAS for MP was performed using PLINK v2.0. A logistic regression framework with an additive genetic model was applied to test the association between each SNP and the phenotype. Because the case–control ratio in our dataset was unbalanced, PLINK’s firth-fallback option was enabled to provide penalized maximum-likelihood estimates when complete or quasi-complete separation occurred. To control for population structure, the top 10 principal components (PC1–PC10) derived from genome-wide genotype data were included as fixed-effect covariates. The final model can be written as:


$$\text{logit}(P(Y_{i}))=\beta_{0}+\beta_{\mathrm{SNP}}*G_{i}+\sum_{k=1}^{10}\beta_{k}*PC_{\mathrm{ik}}$$

Where $Y_{i}$ is the case–control status of individual i, $G_{i}$ is the SNP genotype coded additively as 0,1, or 2 copies of the minor allele, $PC_{\mathrm{ik}}$ represents the kth principal component of individual i, and $\beta_{k}$ coefficients represent the estimated fixed effects. Firth penalized logistic regression was automatically applied to SNPs for which the standard logistic regression failed to converge. Only autosomal chromosomes were retained for GWAS after recoding to a sheep-specific 1–26 chromosome set. Additionally, a suggestive significance threshold was set at *p* < 1.0 × 10^−5^ (15). LD structure was visualized using LDBlockShow based on SNPs extracted from the GWAS-associated regions.

### 2.6 Gene annotation

Genomic annotation of suggestive SNPs was performed using the Ensembl *Ovis aries* reference genome (Oar_v3.1; release 114). The corresponding GTF annotation file (Ovis_aries. Oar_v3.1.114.gtf) was used to obtain genomic coordinates for all annotated genes. SNP chromosomal positions were first standardized to match the reference genome. For each SNP, its genomic position was compared with annotated gene intervals to determine whether it was located within a gene. SNPs falling inside gene boundaries were assigned directly to the corresponding gene. For intergenic SNPs, all genes located within a ± 200 kb window centered on the SNP position were retrieved and recorded. Due to the limited number of annotated candidate genes near the significant SNPs, conventional GO and KEGG analyses had low statistical power. In addition, 51.9% of the genes in this study were uncharacterized. Therefore, we did not rely on enrichment statistics. Instead, we performed manual biological interpretation of individual genes based on known craniofacial developmental mechanisms.

## 3 Results

### 3.1 Results of the phenotypic analysis

A total of 221 Duolang sheep were included in the genome-wide association study, consisting of 182 individuals with the prognathism phenotype and 39 phenotypically normal controls (Figure 1).

### 3.2 Resequencing and sequencing depth results

**Figure 2:**
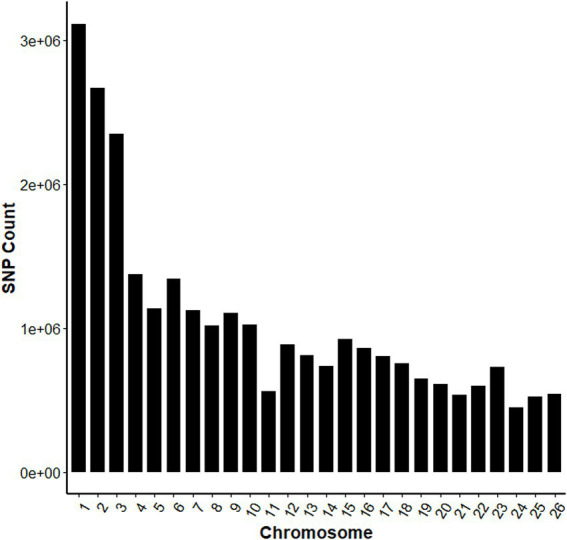
Distribution of SNP counts across chromosomes.

The whole-genome sequencing yielded 6,837.92 Gb of raw sequence data. After stringent quality filtering, 6,780.34 Gb of high-quality clean reads were obtained, with mean Q20 and Q30 values of 96.985 and 92%, respectively. The average GC content was 43.58%, and the mean sequencing depth was approximately 11×. After quality control, one sample was removed due to missing genotype data (−-mind), 220 individuals and 27,172,983 SNPs were retained for subsequent analyses. The SNP numbers for each chromosome are shown in Figure 2.

### 3.3 Principal component analysis (PCA)

**Figure 3:**
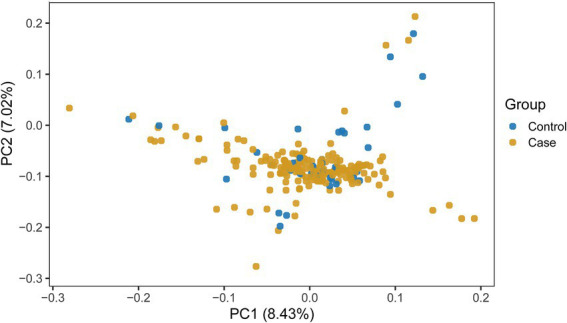
PCA plot.

PCA was performed to assess population structure. The analysis did not reveal distinct clustering of individuals, suggesting minimal stratification within the analyzed samples. The first two principal components explained 8.43 and 7.02% of the genetic variance, respectively (Figure 3). As mentioned above, we nevertheless included the first 10 principal components as covariates in the GWAS model to account for potential subtle population structuration.

### 3.4 Genome-wide association study results

**Figure 4:**
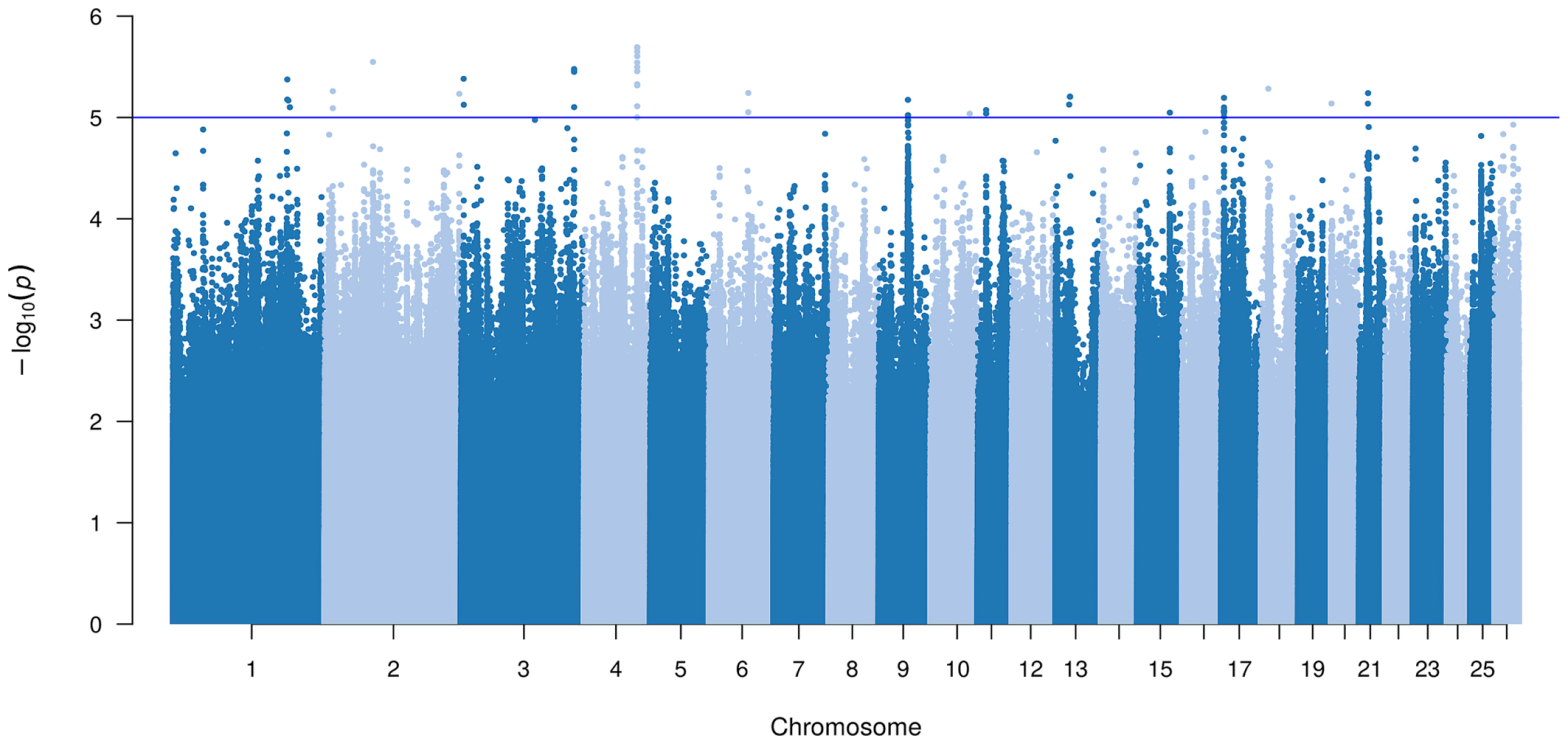
Manhattan plots indicate the suggestive threshold for the GWAS of MP trait.

**Figure 5:**
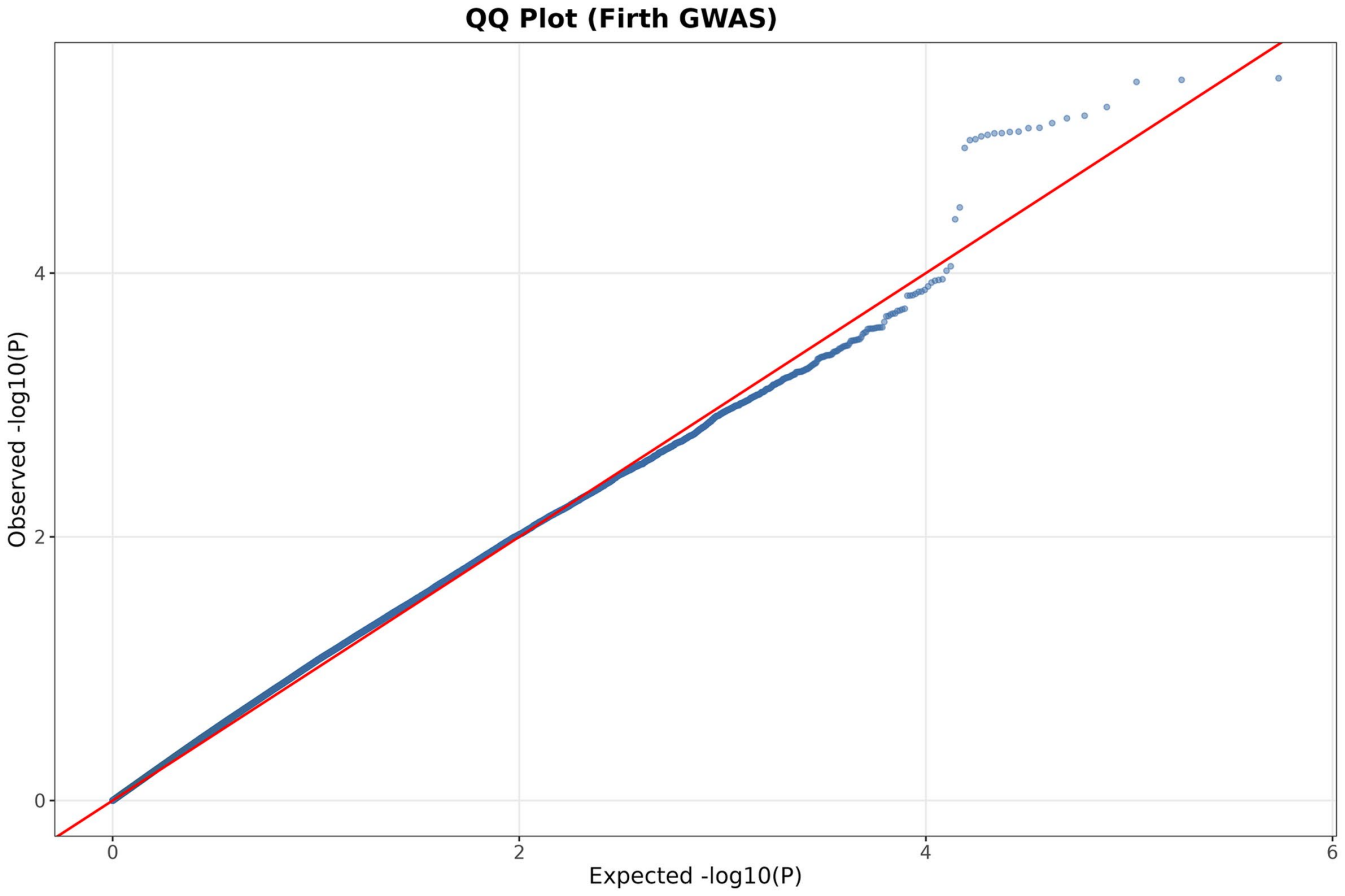
QQplot.

We identified 48 SNPs potentially associated with the trait (Supplementary Table S1). Gene annotation within ±200 kb of these SNPs yielded 77 genes, of which 40 were uncharacterized (Supplementary Table S2). The SNPs were unevenly distributed, with chromosome 4 carrying the most (11 SNPs), followed by chromosome 17 (7 SNPs) (Figure 4). The QQ plot showed that the observed *p*-values closely followed the expected distribution, with a modest deviation in the upper tail. The genomic inflation factor was *λ* = 1.15, indicating acceptable control of population structure and a small number of true association signals (Figure 5).

### 3.5 Regional Manhattan plots and gene annotation

**Figure 6:**
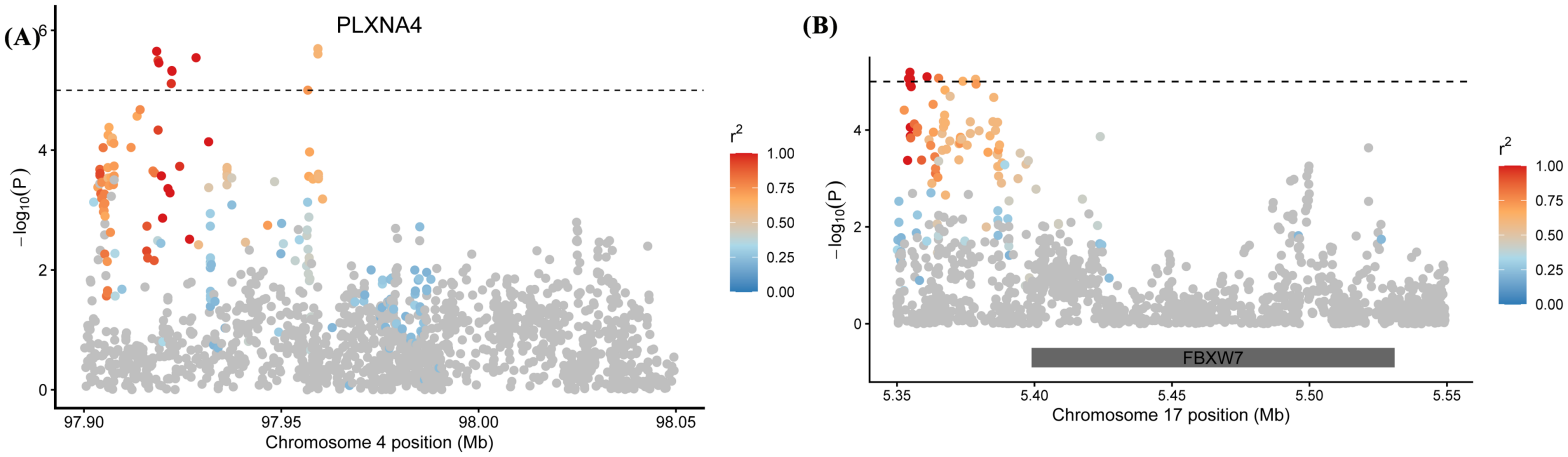
Regional Manhattan plot for the potentially associated region: **(A)** Chromosome 4 and **(B)** Chromosome 17.

To further characterize the significant signals identified in the GWAS, we performed regional association analyses for the top loci on chromosomes 4 and 17 (Figure 6). On chromosome 4 (Figure 6A), we identified a cluster of 11 significant SNPs. The association signals showed a clear peak structure, which mapped directly to the PLXNA4 (Plexin A4) gene region. On chromosome 17 (Figure 6B), a total of 7 significant SNPs were detected. Notably, the genomic interval harboring these variants contains the FBXW7 gene, a key regulator of the Notch signaling pathway.

### 3.6 LD analysis of two region

**Figure 7:**
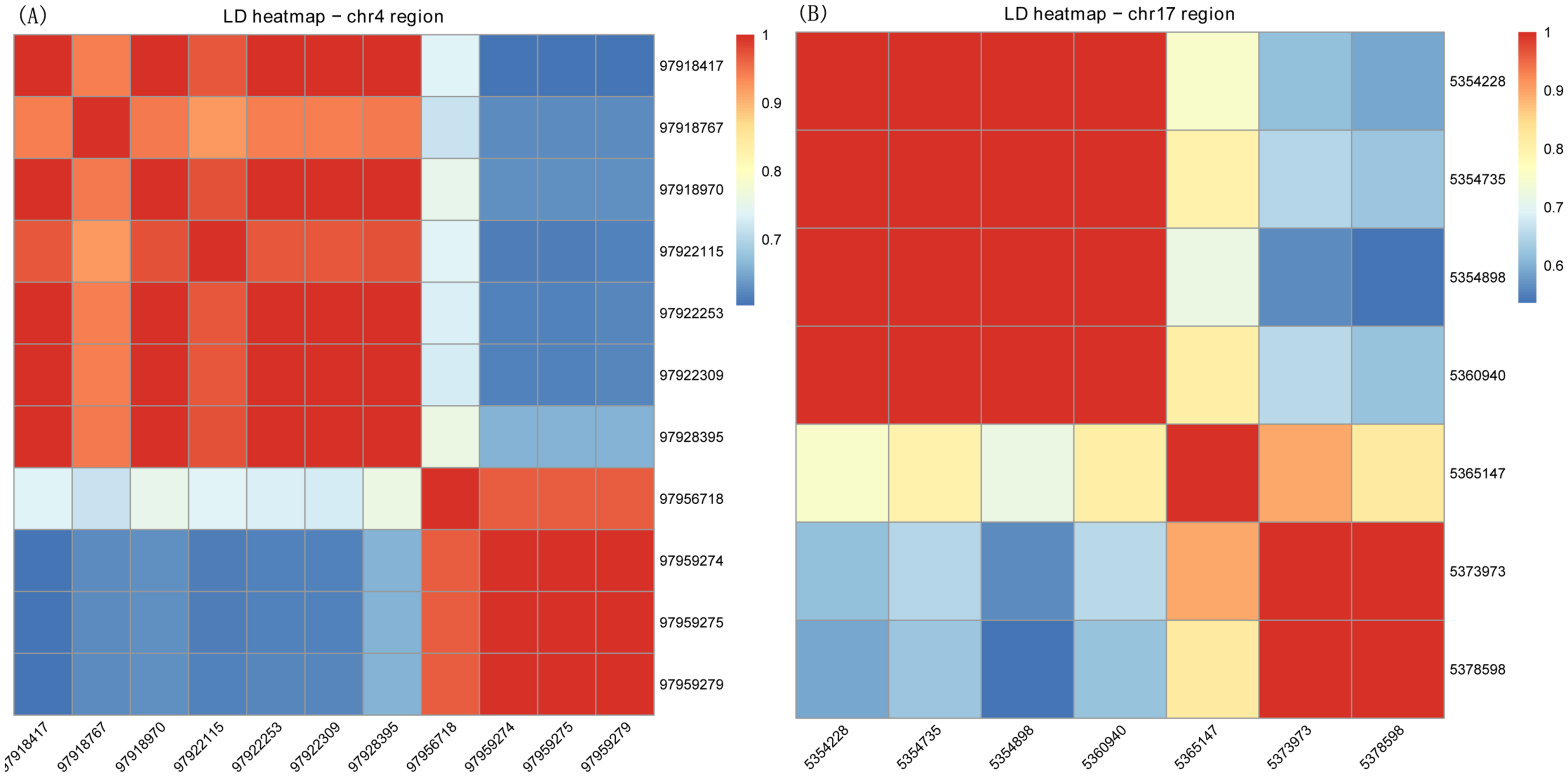
LD heatmap. **(A)** Chr 4 region. **(B)** Chr 17 region. The color scale represents the strength of pairwise LD (r^2^ value), ranging from blue (low LD) to red (high LD). The axes display the positions of the SNPs.

We then computed pairwise LD among the suggestive SNPs within each region and generated LD heatmaps (Figure 7), which revealed the LD structure and confirmed whether the associated SNPs represented a single haplotype block. Pairwise LD analysis among the 11 SNPs on chr4 and among the 7 SNPs on chr17 showed consistently high LD (mean r^2^ = 0.81, range 0.60–1.0 and mean r^2^ = 0.79, range 0.54–1.0, respectively). For the locus on chromosome 4 (Figure 7A), the heatmap revealed a distinct haplotype block structure. The leading SNPs (e.g., from 97,918,417 to 97,928,395) exhibited strong linkage disequilibrium (indicated by the red blocks), suggesting that these variants are inherited together and could therefore potentially tag the same functional mutation within the PLXNA4 gene. Similarly, on chromosome 17 (Figure 7B), a tight LD block was observed among the top associated SNPs (e.g., 5,354,228 to 5,360,940). These high degrees of linkage (r^2^ approaching 1) might contribute to narrow down the genomic interval bearing putative causal variants.

### 3.7 Gene annotation analysis

We annotated the 48 SNPs and identified a total of 77 genes. Twenty SNPs were located within annotated genes, and the remaining SNPs were positioned in upstream or downstream regions. On chromosome 4, seven SNPs were located within the PLXNA4 gene, and four SNPs were located in the intergenic region between PLXNA4 and CHCHD3. On chromosome 17, seven SNPs were located in the intergenic region between TMEM154 and FBXW7. Based on published literature, we classified the annotated genes according to their known functions. These candidate genes can be broadly grouped into four functional categories. Craniofacial, neural crest, and developmental regulators, including PAX7, PLXNA4, CHD2, CHCHD3, FBXW7, DZIP1, CEP135, and KHDRBS2, which are involved in neural crest cell fate, craniofacial patterning, major signaling pathways (e.g., Hedgehog, ERK, Notch), and cytoskeletal organization. Nervous system–related genes, such as GPR158, OPTN, UBR4, MPPED2, EXOC1, and DNAJC3, which participate in neural development, axon guidance, synaptic processes, and cellular stress responses. Epithelial, structural, and metabolic genes, including CLDN10, LAMC3, PLPP7, POMT1, PRRC2B, and AIF1L, associated with cell adhesion, epithelial integrity, extracellular matrix organization, glycosylation, energy metabolism, and cell proliferation. Poorly annotated and tRNA-related genes, such as TRNAC-GCA, and TRNAR-ACG, which remain uncharacterized.

### 3.8 Functional annotation of candidate genes

We searched the literature for the known functions of these genes. Some are involved in craniofacial nerve development. Others are related to the nervous system, or to epithelial cell structure and metabolism. Based on these functions, PAX7 (Paired Box Gene 7), PLXNA4 (plexin-A4), CHD2 (Chromodomain Helicase DNA Binding Protein 2), DZIP1 (DAZ interacting zinc finger protein 1) and FBXW7 (F-box and WD repeat domain-containing 7) are the most likely candidates for mandibular prognathism in Duolang sheep. PAX7 is a transcription factor belonging to the PAX gene familyand is characterized by a paired DNA-binding domain. It plays important roles in muscle formation, neural development, and craniofacial morphogenesis. Functional studies have demonstrated that PAX7 contributes to neural crest specification and craniofacial patterning. In chicken embryos, Basch et al. reported that PAX7 is required for neural crest specification during gastrulation (24). In mammals, Mansouri et al. reported that PAX7 knockout in mice leads to aberrant craniofacial neural crest cell development (25). Further, Cates et al. applied particle-based shape modeling to compare cranial base morphology between Pax7-deficient and wild-type neonatal mice, and identified systematic alterations such as increased cranial base width and anterior–inferior bending of the posterior cranial margin (26). Together, these studies indicate that PAX7 is involved in the regulation of cranial neural cell fate and craniofacial development. Mechanistically, its lineage descendants are widely incorporated into cranial neural crest–derived structures, including cranial cartilage, suggesting that disruption of PAX7 may cause abnormal cranial and mandibular development (27), In humans, PAX7 has been reported to be associated with mandibular prognathism (28).

CHD2 is a member of ATP-dependent CHD chromatin remodeling family (29, 30). The canonical function of CHD2, like other chromatin remodelers, is to regulate chromatin accessibility (31). Early evidence from a chromosomal deletion study proposed that CHD2 loss may contribute to congenital anomalies, with skeletal abnormalities noted among affected individuals (32). Subsequent clinical reviews have expanded the phenotypic spectrum, documenting patients with CHD2 variants who exhibit growth disturbances, skeletal or physical abnormalities, and, in some cases, subtle facial dysmorphism (33). Case-based studies similarly describe variable morphological findings, including craniofacial or growth-related anomalies, although such features are inconsistent and typically secondary to neurological presentations (34). Broader analyses of CHD2-related microdeletions further highlight substantial phenotypic variability, encompassing skeletal, muscular, and craniofacial manifestations across different patients (35). Previous studies have reported that variants in CHD2 are associated with developmental disorders.

DZIP1 encodes a zinc-finger protein originally identified in zebrafish as the *iguana* locus and acts as a core regulator of primary ciliogenesis and Hedgehog (Hh) signal transduction (36, 37). Subsequent studies established DZIP1 as an essential component of the ciliogenic pathway, required for axonemal biogenesis and basal-body function (38). To date, no studies have directly linked *DZIP1* or its paralog *DZIP1L* to mandibular prognathism in humans or animal models. *DZIP1* is a regulator of primary ciliogenesis and Hedgehog signaling (39), both of which play critical roles in cranial neural crest differentiation and craniofacial morphogenesis. Loss of *dzip1* in zebrafish results in ocular coloboma and abnormal cranial neural crest–derived tissue patterning (40). These findings support a role for *DZIP1* in craniofacial developmental processes.

## 4 Discussion

### 4.1 Genetic basis of mandibular prognathism in Duolang sheep

Similar domestication-driven selection on standing variation has been documented in horses, based on ancient genome time-series analyses of regulatory loci linked to behavior and body conformation (41), paralleling our findings that genetic variants affecting craniofacial morphology may have been targets of selection in domesticated sheep. In this study, we performed a GWAS using the presence or absence of MP in Duolang sheep as the phenotype. A total of 220 individuals were examined, potentially providing sufficient statistical power to detect effects of reasonable size. Defining the trait as binary simplified the complex craniofacial features. It also allowed clear distinction between individuals with different morphologies. With rigorous control of population structure and stringent statistical thresholds, we obtained reliable association signals. Notably, significant regions were detected on chromosomes 4 and 17, suggesting that MP has a clear molecular basis. A study in Scottish Blackface sheep showed that feeding level and pasture type can affect some dental traits, the authors noted that feeding conditions may change eruption timing or tooth size, but they do not meaningfully alter occlusal relationships, especially the anteroposterior jaw position (42). Taken together, these observations suggest that while environmental conditions may modulate dental characteristics, mandibular prognathism itself is more likely to reflect underlying developmental and genetic mechanisms rather than postnatal environmental effects.

The identification of a limited set of candidate genes in our GWAS is consistent with this characteristic. Several candidate genes detected in this study overlap with known craniofacial developmental genes in humans and mice, which might add support to our findings. MP is not only an anatomical feature but may also influence feeding efficiency, mastication, and overall health in animals. The key genes identified in this study could serve as potential molecular markers for future breeding programs. In addition, our results provide genetic evidence for the evolution of jaw morphology in livestock. Since some candidate genes identified here are also implicated in craniofacial development in humans and mice, this suggests that mandibular prognathism in sheep shares conserved genetic mechanisms with jaw development in other mammals.

### 4.2 Functional interpretation of PLXNA4 and FBXW7 identified by GWAS

Among the candidate genes identified in this study, PLXNA4 and FBXW7 stand out as biologically coherent and may be highly relevant to mandibular prognathism, as both genes participate in conserved developmental pathways that link neural patterning, tissue organization and skeletal growth across vertebrate species. PLXNA4 encodes the semaphorin receptor plexin-A4, a central component of the SEMA3A–NRP1–PLXNA4 signaling axis that governs axonal guidance and cranial nerve organization (43, 44). Functional evidence from multiple model organisms highlights the evolutionary conservation of this pathway. In mice, loss of PLXNA4 leads to defasciculation of cranial branchiomotor nerves, including facial nerves such as the greater superficial petrosal nerve (GSPN), resulting in aberrant midline crossing due to disruption of SEMA3A–NRP1–PLXNA4 signaling. Additional studies have shown that plexin-A4 is expressed in the ventral posteromedial nucleus (VPM) of the thalamus, where its deficiency disrupts the organization of thalamocortical projections, highlighting its essential role in cortical somatosensory patterning (45). In zebrafish embryos, loss of plexin-A4 reduces the branching of Rohon–Beard sensory neurons and trigeminal ganglion axons, suggesting that plexin-A4 also promotes axonal branching and elaboration (46). Collectively, these studies indicate that PLXNA4 is involved in semaphorin-mediated axonal guidance and neuronal patterning. The identification of a strong association signal spanning PLXNA4 in Duolang sheep therefore suggests that perturbation of conserved axon guidance pathways may contribute to variation in mandibular architecture.

FBXW7 (or hCdc4), a substrate-recognition component of the SCF ubiquitin ligase complex, represents a second compelling candidate linking developmental regulation to mandibular structure (47, 48). Many studies on this gene are related to cancer (20, 49, 50). In addition to its role in tumorigenesis, FBXW7 has been implicated in multiple developmental processes. In mesenchymal progenitor cells, FBXW7 has been shown to control osteogenic and chondrogenic differentiation by targeting the endoplasmic reticulum–anchored transcription factors OASIS and BBF2H7 for proteasome-mediated degradation, and conditional deletion of FBXW7 in mouse mesenchymal cells promotes both osteogenesis and chondrogenesis *in vitro* (51). In articular cartilage, FBXW7 has also been implicated in cartilage homeostasis and osteoarthritis: mechanical overloading or ageing leads to reduced FBXW7 expression in chondrocytes, which is associated with enhanced chondrocyte senescence, increased catabolic marker expression and accelerated cartilage degeneration in mouse models (52, 53). Additional work in osteoarthritis models indicates that FBXW7 can modulate chondrocyte degeneration at least in part through regulation of the HIF-1α/VEGF axis (54). Germline loss-of-function variants in FBXW7 cause a multisystem neurodevelopmental syndrome characterized by intellectual disability, hypotonia and structural brain anomalies (55). Taken together, these findings suggest that FBXW7 may influence mandibular prognathism through modulation of chondrogenesis, cartilage maintenance and growth dynamics of the mandibular skeleton.

Collectively, the identification of PLXNA4 and FBXW7 in this GWAS points to a developmental framework in which neuromuscular patterning and osteochondral regulation act in concert to shape mandibular morphology.

### 4.3 Integration of candidate pathways underlying mandibular prognathism in Duolang sheep

The mandible derives from cranial neural crest–derived mesenchyme of the first pharyngeal (mandibular) arch, and develops through Meckel’s cartilage and subsequent ossification (56), these processes are orchestrated by a network of signalling pathways, including Hedgehog (Hh), BMP/FGF, Notch, semaphorin–plexin guidance cues, which together control CNC survival, patterning and osteochondrogenic differentiation during craniofacial morphogenesis.

The Notch signalling pathway is associated with mandibular prognathism and related craniofacial morphology (57, 58). NOTCH3 is one of the substrates of FBXW7 (59). Loss or mutation of FBXW7 can lead to sustained activation of Notch signalling (60). Therefore, we hypothesize that FBXW7 may regulate mandibular growth through the Notch signalling pathway.

Our candidates may be conceptualized as lying along a single developmental axis in which PAX7 and CHD2 are thought to influence the specification and epigenetic priming of cranial neural crest cells, DZIP1-dependent ciliary HH signaling may help sustain their survival and early mandibular patterning, PLXNA4-mediated Semaphorin–Plexin cues are likely to contribute to the neuromuscular loading environment experienced by the jaw, and FBXW7-dependent control of Notch and cell-cycle dynamics in osteochondral cells may modulate matrix production and remodeling. Within such a multi-layered CNCC–HH–neuromuscular–osteogenic framework, small allelic effects at each node could act in concert to bias mandibular growth vectors, potentially offering a biologically plausible explanation for the polygenic associations detected in our GWAS.

### 4.4 Limitations

Due to the lack of mandibular bone samples, GWAS-identified candidate genes could not be experimentally validated, and gene-level mechanistic interpretations therefore remain provisional. To address this, future work will prioritize sheep-focused validation, including use a quantitative measure rather than a binary one, improve the statistical model, use haplotypes and spatial expression mapping in embryonic mandibular tissues (e.g., *in situ* hybridization or immunolabeling), perturbation studies in ovine cells or embryos (e.g., targeted editing within ethical and welfare constraints), and larger population genetic analyses with fine-mapping and replication.

## 5 Conclusion

This study provides new insights into the genetic basis of MP in Duolang sheep. We identified five genes that may be associated with MP trait. Future studies should focus on functional validation of these candidate genes, investigation of gene–environment interactions, and cross-validation in different populations to further elucidate the complex genetic mechanisms underlying MP.

## Data Availability

The datasets presented in this study can be found in online repositories. The names of the repository/repositories and accession number(s) can be found at: https://identifiers.org/ncbi/insdc.sra:SRP544918 and https://identifiers.org/ena.embl:PRJEB83806.
