# Supplementary material for: Identification of key genes for mandibular prognathism in Duolang sheep via genome-wide association analysis
Source: Front Vet Sci. 2026 Jan 19;12:1719178. doi: 10.3389/fvets.2025.1719178 (PMC12862929; doi:10.3389/fvets.2025.1719178)
Supplement: Supplementary file 2 [file Table_2.DOCX]

| **Table S1 **Distribution of Candidate SNPs**** | | | | | | |
| --- | --- | --- | --- | --- | --- | --- |
| #CHROM | POS | REF | ALT | A1 | TEST | P |
| 1 | 210181901 | C | T | T | ADD | 6.65575e-06 |
| 1 | 210181937 | G | A | A | ADD | 4.21743e-06 |
| 1 | 212081497 | C | T | T | ADD | 6.80332e-06 |
| 1 | 214919355 | TC | T | T | ADD | 7.9244e-06 |
| 2 | 15174936 | G | A | G | ADD | 8.0941e-06 |
| 2 | 88914054 | A | G | G | ADD | 2.83005e-06 |
| 2 | 247449457 | C | T | T | ADD | 5.84723e-06 |
| 2 | 15181231 | G | A | A | ADD | 5.50405e-06 |
| 3 | 5288045 | C | G | G | ADD | 4.15256e-06 |
| 3 | 5512487 | G | T | T | ADD | 7.49293e-06 |
| 3 | 208188693 | C | T | T | ADD | 3.32768e-06 |
| 3 | 208188694 | T | A | A | ADD | 3.42391e-06 |
| 3 | 208188697 | C | T | T | ADD | 3.54818e-06 |
| 3 | 208188699 | G | T | T | ADD | 7.90718e-06 |
| 4 | 97918417 | C | T | T | ADD | 2.24224e-06 |
| 4 | 97918767 | G | T | T | ADD | 3.17952e-06 |
| 4 | 97918970 | A | G | G | ADD | 3.49931e-06 |
| 4 | 97922115 | A | C | C | ADD | 7.7455e-06 |
| 4 | 97922253 | G | A | A | ADD | 4.69887e-06 |
| 4 | 97922309 | A | G | G | ADD | 4.82769e-06 |
| 4 | 97928395 | C | T | T | ADD | 2.86646e-06 |
| 4 | 97956718 | G | A | A | ADD | 9.98462e-06 |
| 4 | 97959274 | A | G | G | ADD | 2.05731e-06 |
| 4 | 97959275 | A | C | C | ADD | 2.02388e-06 |
| 4 | 97959279 | C | T | T | ADD | 2.48404e-06 |
| 6 | 72158312 | T | C | C | ADD | 5.7313e-06 |
| 6 | 72160723 | A | G | A | ADD | 8.87145e-06 |
| 9 | 53995983 | G | A | A | ADD | 9.48401e-06 |
| 9 | 53876761 | G | GA | GA | ADD | 6.6981e-06 |
| 9 | 53878689 | T | C | C | ADD | 9.65235e-06 |
| 10 | 72214393 | C | T | T | ADD | 9.1841e-06 |
| 11 | 15910724 | G | A | A | ADD | 9.1638e-06 |
| 11 | 15874271 | A | T | T | ADD | 8.45269e-06 |
| 13 | 25472136 | T | C | C | ADD | 7.46942e-06 |
| 13 | 27005450 | A | G | A | ADD | 6.23509e-06 |
| 13 | 27005451 | G | A | G | ADD | 6.23509e-06 |
| 15 | 60383071 | T | G | G | ADD | 8.95926e-06 |
| 17 | 5354228 | T | G | G | ADD | 8.67429e-06 |
| 17 | 5354735 | C | T | T | ADD | 6.40243e-06 |
| 17 | 5354898 | A | G | G | ADD | 8.70878e-06 |
| 17 | 5360940 | T | C | C | ADD | 7.95516e-06 |
| 17 | 5365147 | T | C | C | ADD | 8.51117e-06 |
| 17 | 5373973 | C | T | T | ADD | 9.81745e-06 |
| 17 | 5378598 | AAGT | A | A | ADD | 8.94748e-06 |
| 18 | 13661439 | C | T | T | ADD | 5.2042e-06 |
| 20 | 1093567 | A | G | G | ADD | 7.28348e-06 |
| 21 | 16519913 | G | A | G | ADD | 7.30252e-06 |
| 21 | 16823471 | AC | A | A | ADD | 5.75056e-06 |
